# Chasing Zero Harm in Radiation Oncology: Using Pre-treatment Peer Review

**DOI:** 10.3389/fonc.2019.00302

**Published:** 2019-04-24

**Authors:** Srinivasan Vijayakumar, William Neil Duggar, Satya Packianathan, Bart Morris, Chunli Claus Yang

**Affiliations:** Radiation Oncology Department, University of MS Medical Center, Jackson, MS, United States

**Keywords:** pre-treatment peer review, chasing zero harm, quality assurance, safety in radiation treatment, radiation oncology

## Abstract

**Purpose:** The Joint Commission has encouraged the healthcare industry to become “High Reliability Organizations” by “Chasing Zero Harm” in patient care. In radiation oncology, the time point of quality checks determines whether errors are prevented or only mitigated. Thus, to “chase zero” in radiation oncology, peer review has to be implemented prior to treatment initiation. A multidisciplinary group consensus peer review (GCPR) model is used pre-treatment at our institution and has been successful in our efforts to “chase zero harm” in patient care.

**Methods:** With the GCPR model, policy-defined complex cases go through a treatment planning conference, which includes physicians, residents, physicists, and dosimetrists. Three major plan aspects are reviewed: target volumes, target and normal tissue dose coverage, and dose distributions. During the review, any team member can ask questions and afterwards a group consensus is taken regarding plan approval.

**Results:** The GCPR model has been implemented through a commitment to peer review and creative conference scheduling. Automated analysis software is used to depict color-coded results for department approved target coverage and dose constraints. About 8% of plans required re-planning while about 23% required minor changes. The mean time for review of each plan was 8 min.

**Conclusions:** Catching errors prior to treatment is the only way to “chase zero” in radiation oncology. Various types of errors may exist in treatment plans and our GCPR model succeeds in preventing many errors of all shapes and sizes in target definition, dose prescriptions, and treatment plans from ever reaching the patients.

## Background and Introduction

Since the release of the seminal report from the Institute of Medicine (IOM) titled, “To Err is Human,” in 1999, the importance of patient safety and avoiding medical errors have been recognized and endorsed by the physician community, as well as by governing bodies and accreditation agencies (1). Stelfox et al. (2) assessed the impact of the initial IOM report and reported that it had increased the number of patient safety-related publications and awards. While a total of 5,514 articles were published over a 10 year period until 2006, the rate nearly tripled from 59 to 164 manuscripts/100,000 Medline citations following the publication of the IOM monograph. More importantly, it started a movement to emphasize the importance of a safety-oriented culture in patient care in the USA and beyond (3). A follow-up IOM report titled, “Crossing the Quality Chasm,” addressed additional quality issues and defined the six focal points of safety in patient care:

1. Safe care.
2. Effective care.
3. Patient-centered care.
4. Timely care.
5. Efficient care.
6. Equitable care.

In light of these hard hitting IOM reports the US Federal Government took a substantial interest in improving patient safety and its efforts have been detailed in a recent publication which emphasizes that there is no magical means of elimination of all medical errors; it argues, however, that the following strategies when systematically applied, improve overall patient outcomes by avoiding patient harm (4):

- Not tolerating high error rates.
- Setting ambitious targets for error reduction initiatives.
- Developing tracking mechanisms that expose errors.
- Relying on abundant reports of “errors” and “near misses.”
- Thoroughly investigating errors, including performing root causes analyses.
- Applying to error reduction a systems approach that embraces a wide array of human factor, technical, and organizational remedies.
- Focusing on systems solutions that do “*not*” seek to impart individual fault and blame.
- Changing an organizational culture so that it embraces safety and error reduction.
- Allocating adequate resources to error prevention initiatives and the development of the knowledge bases to support them.
- Recognizing that solutions often come from unexpected sources; i.e., from “out of the box” thinking and new combinations of established disciplines (e.g., human factors psychology with aeronautical engineering).

The impetus behind these developments has evolved into a re-imagined undertaking and systematic improvement process spear-headed by the Joint Commission for Accreditation of Healthcare Organizations (JCAHO) called, “Chasing Zero Harm” (5–8). This campaign aims to transform healthcare entities into high reliability organizations (HROs) moving toward the goal of zero patient harm (9). In the quest to achieve “zero harm” by becoming a HRO, not only would the risk of harm from healthcare interventions be reduced, there will also be the “collateral” benefits of a more efficacious and efficient use of healthcare resources (10). It is inspiring to note that the “chasing zero harm” movement is also making an impact world-wide in improving patient safety and their well-being (11).

As we review more extensively in the Discussion section, radiation oncology is a highly intricate field; and the software and hardware technology underlying radiation treatment planning and delivery has become significantly complex in the past two decades, thereby increasing the probability of a planning or treatment error (12). While radiation oncology already has a long history and a solid reputation for safety (13–15), some of the quality assurance and review processes in place may be inadequate to truly “chase zero” and their improvements can only have an additional beneficial impact on the patients we serve. In that spirit, we recommend the use of a pre-treatment and multidisciplinary group consensus peer review (GCPR) program in radiation oncology, discuss our experience with its use, and encourage its wider adoption and further improvement by the radiation oncology community.

### Our Institutional Model

In order to accomplish high-quality pre-treatment peer review in our clinic, a group-consensus model that has shown efficacy in the radiology environments has been adapted (16). A treatment planning conference has been specifically designed to include at a minimum two physicians besides the attending physician of the case to be reviewed, plus other members of the traditional divisions in a radiation therapy clinic such as physics, dosimetry, physician/physics residents, and even therapy when possible. Cases are screened by attending physicians for presentation at the conference for the rigid criteria of IMRT, VMAT, stereotactic body radiation therapy (SBRT), pediatric, re-irradiation treatment, and new modalities/techniques, with the inclusion of additional cases based on physician preference and in consultation with physics and dosimetry when needed. All of these cases are sent to conference only AFTER the treatment plan has been reviewed and approved by the attending physician, but BEFORE the plan has been processed for treatment and plan verification *via* standard QA practices (17, 18).

**Figure 1:**
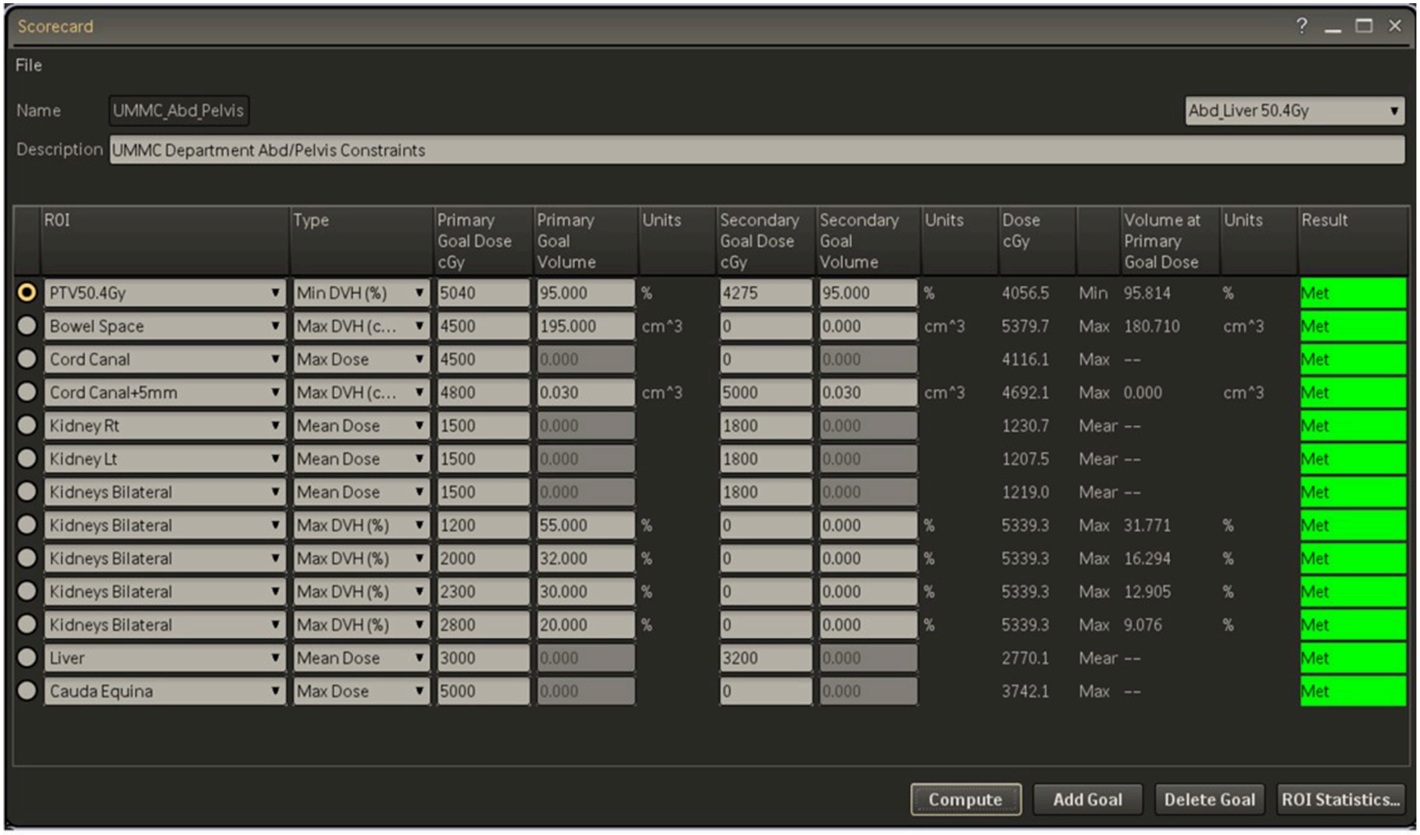
Example of automated DVH analysis using Pinnacle Scorecard.

**Figure 2:**
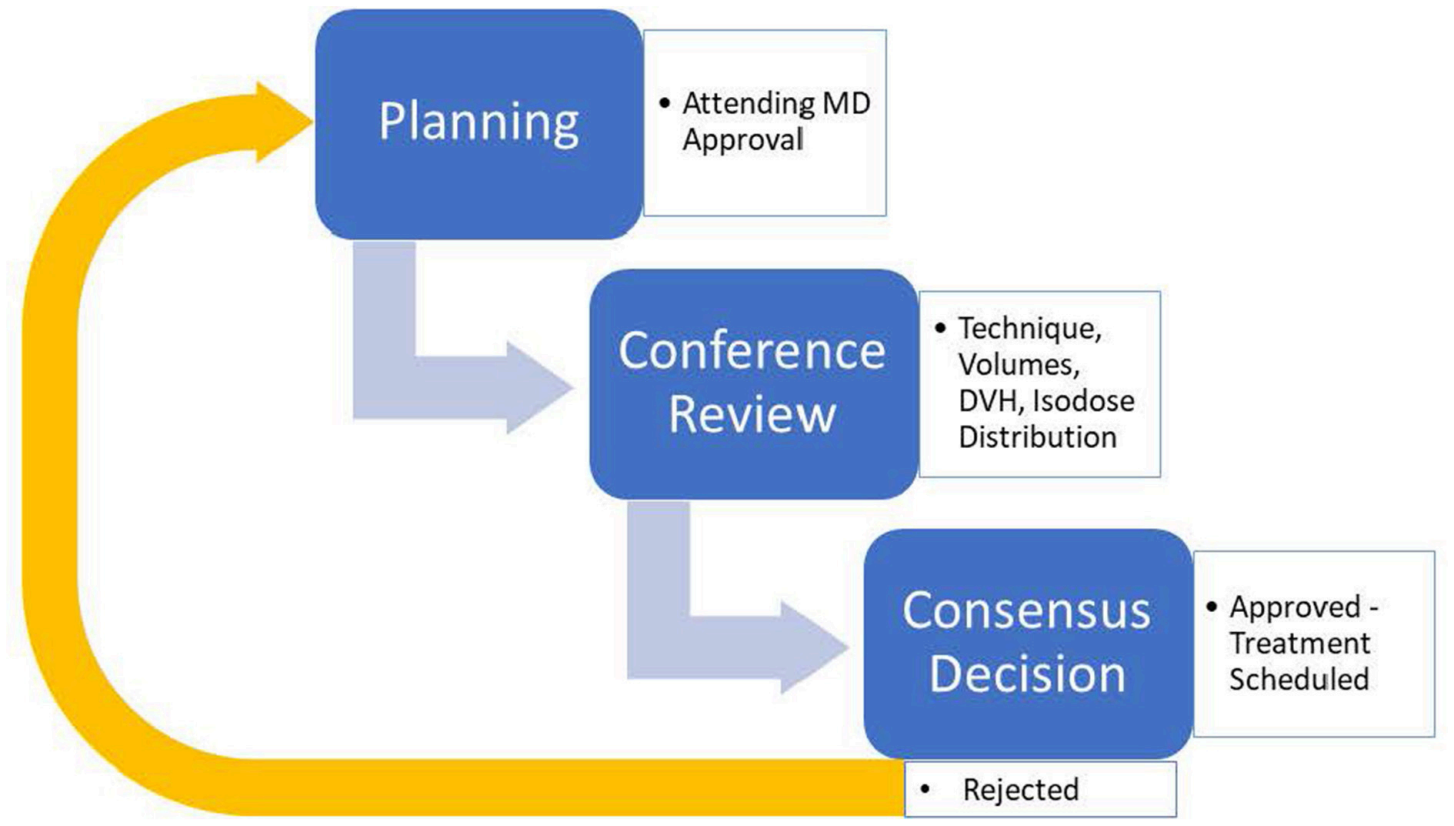
General description of the Group Consensus Pre-Treatment Peer review model in implementation.

During the presentation, each case is reviewed in a basic four-step process. Each case is first introduced with a brief case history including diagnosis and staging. Next, target volumes are reviewed, specifically GTV and its expansions to CTV and subsequently to PTV. Image fusions and 4D-CT scans are also included in the review at this step for applicable cases. Third, an automated dose-volume-histogram (DVH) analysis tool within the Pinnacle™ treatment planning system (V16.2, seen in Figure 1) is used to highlight plan target coverages and doses to critical structures based on our group-approved and/or nationally endorsed tissue tolerances and coverage levels (18). Anyone can request that the actual DVH curves be reviewed if any questions or concerns exist after this initial “Scorecard” review. Finally, the isodose lines relative to the target volumes are reviewed including the location of the maximum dose and the overall distribution of isodoses relative to targets and normal tissues. During the review, any team member may inquire about technical or clinical aspects of the plan to improve treatment decision-making. Once the review and any additional commentary/questions have been addressed, an open vote is taken by all present attending physicians in regards to plan approval. Based on the vote, a plan will be designated as approved, approved with minor changes such as a fraction size or number change, or disapproved with a need for re-planning with a specified reason (17). The general design can be seen in Figure 2.

Over a period of about 11 weeks, 73 cases were reviewed using our GCPR model and the rate of plan changes and presentation time was prospectively recorded in addition to important plan parameters. Major changes were defined as changes requiring re-planning in dosimetry and representation at conference while minor changes were those that could be applied during plan processing without re-planning such as a fraction or dose change (19).

### Our Institutional Experience

Based on our published data, about 8.2% of plans undergo some type of re-planning and 23.3% proceed with approval and minor changes; the rest were approved outright. Most plans were presented within the 10 min time frame allocated per plan while the mean time per plan presentation was 8 min (19).

This model has been viable at our institution for various reasons, including a collective commitment to *pre*-treatment peer review and appropriate handling of the clinical schedule. One hour is blocked off twice a week in the morning for all physicians to be able to attend these conferences. To avoid unnecessary delay in initiating treatment, *ad hoc* conferences are sometimes held during lunch hours to handle overflow patients or those which could not be presented at the normal conference time (17). Electronic tools such as the “Scorecard” are used where possible to provide useful information in an efficient and easy to interpret manner (18).

## Discussion

### High Reliability Organizations and Chasing Zero Harm

To the best of our knowledge, it appears that the actor Dennis Quaid, who made the documentary, “Chasing Zero: Winning the War on Healthcare Harm,” may have popularized the phrase, “Chasing Zero Harm” (20). However, the phrase probably originated at a 2008 medical conference on “Hospital Acquired Infections” (6). We propose, however, that credit for the beginnings of the “Zero Harm Movement” in healthcare should be attributed to the classic article by Chassin and Loeb (21). In an earlier manuscript in “Health Affairs,” these authors had traced the history of the attempts to reduce harm in healthcare and presented the conclusion that the way to maximally prevent harm was to make healthcare organizations “Highly Reliable Organizations” (HROs) (22). In their latter paper (2013), the authors described what it would take for a healthcare organization to become a “Highly Reliable” entity in regards to patient safety. Illustrating how other industries achieved their “high reliability” status (the airline and nuclear power plant industries for example), they issued a call to action to healthcare organizations to aim high to reach loftier levels of patient safety (i.e., pursuing “zero harm”). However, “chasing zero harm” cannot happen in a vacuum; Chassin and Loeb also emphasized the qualities of the mature HRO that achieves systematic harm reduction goals (Table 1) (21). These qualities can be summarized into 3 major categories:

1. Leadership that is committed to chasing zero harm.
2. A pervasive safety culture in the organization and an aim to achieve such a culture.
3. Wide deployment of effective process improvement tools.

**Table 1:** Qualities of the mature HRO that achieves systematic harm reduction goals.

**Characteristics of successful and mature High Reliability Organizations [HROs] (21)**
1. Quality is the organization's highest-priority strategic goal.
2. Key quality measures are routinely displayed internally and reported publicly.
3. Reward systems for staff prominently reflect the accomplishment of quality goals.
4. Safely adopted IT solutions are integral to sustaining improved quality.
5. High levels of (measured) trust exist in all clinical areas; self-policing of codes of behavior is in place.
6. All staff recognize and act on their personal accountability for maintaining a culture of safety.
7. Equitable and transparent disciplinary procedures are fully adopted across the organization.
8. Close calls and unsafe conditions are routinely reported, leading to early problem resolution before patients are harmed; results are routinely communicated.
9. System defenses are proactively assessed, and weaknesses are proactively repaired.
10. Safety culture measures are part of the strategic metrics reported.
11. Systematic improvement initiatives are under way to achieve a fully functioning safety culture.
12. Adoption of RPI [Robust Process Improvement] tools is accepted fully throughout the organization.
13. Training in RPI is mandatory for all staff, as appropriate to their jobs.
14. RPI tools are used throughout the organization for all improvement work; patients are engaged in redesigning care processes.

How can we further understand the concept of a HRO? Baker et al. (23) define HROs as highly complex entities which, “exist in such hazardous environments where the consequences of errors are high, but the occurrence of error is extremely small”. In addition, HROs are entities that have a, “Collective Mindfulness,” as described by Weick and Sutcliffe (24) (see Table 2), as they corporately practice a constant vigilance focused on preventing rather than reacting to errors. A strong component of the vigilance in these organizations is the, “Robust Process Improvement,” (RPI) program—an essential component of the institution's safety culture that permeates all aspects of its activities. Although a full description of RPI programs is beyond the scope of this communication, a brief reference to its components is made in Table 3 (25).

**Table 2:** Collective Mindfulness as described by Weick and Sutcliffe (24).

**Qualities in a “Collective Mindfulness” organization**
1. As individuals and as a team, everybody cares about safety; it is something on the top of their mind always.
2. They are aware that even small deviations from normal protocols / processes can lead to catastrophic consequences.
3. They are continuously on the lookout for small or large deviations from routine and pay attention to them with the fear that these deviations are the initials signs of major error that could happen and that could be prevented.
4. Such continuous “surveillance” leads these organizations to prevent rather than react to errors.

**Table 3:** Components of Robust Process Improvement.

**A brief outline of Robust Process Improvement [RPI](25)**
A process is robust when it consistently achieves high quality in the following ways:
• Recognizing and seeking the voice of the staff.
• Defining factors critical to quality.
• Using data and data analysis to design improvement.
• Enlisting stakeholders and process owners in creating and sustaining solutions.
• Eliminating defects and waste.
• Drastically decreasing failure rates.
• Simplifying and increasing the speed of processes.
• Partnering with staff and leaders to seek, commit to, and accept change.
The RPI tool kit includes methodologies such as Lean Six Sigma, Facilitating Change, and Work Out.

Outside of the airline or nuclear power plant industries, a perfect example of an entity that needs to be a HRO is any radiation oncology department. Radiation oncology is a specialty where the likelihood of any major error is negligible, yet where the consequences of such error can be catastrophic to a patient. In this paper, we examine the underlying premises and shortcomings of some of the established quality assurance activities that currently underpin safety in radiation oncology departments and argue for the inclusion of a robust pre-treatment peer review program as part of a RPI in radiation oncology. We begin with a brief overview of safety in radiation oncology.

### Ensuring Safety in Radiation Oncology

As a specialty, radiation oncology has a long history and a solid reputation for safety (13–15). The gravity of complications associated with ionizing radiation overdoses were recognized early and the radiation oncology community has been very diligent in its quality assurance efforts to minimize such events. As a result of this vigilance, radiation dose errors and misadministration events are quite rare occurrences. In a 4-year review of such errors at a major academic institution, Das et al. (26) found only a 0.66% error rate for treated patients and a 0.03% event-rate for number of fractions of radiation therapy; overall, there were 358 near misses among 28,488 new patients producing an error rate of ~1.3%. About 80% of these were minor errors. Hunt et al. (27) found similar error rates: 0.93% per course and 0.05% per treatment session. They also reported a declining trend over a 10-year period in the error rates as computerization of treatment delivery, improvement in treatment technology, and more robust treatment record-and-verify systems were installed. In a comparison of errors between Intensity Modulated Radiation Therapy (IMRT) vs. 3D/conformal RT (3DCRT) in the Harvard Radiation Oncology System, Margalit et al. (28) also found low error rates (155 errors in 241,546 treatment fractions or 0.06%).

Radiation oncology is a highly intricate field and the software and hardware technology underlying radiation treatment planning and delivery has become significantly complex in the past two decades, thereby increasing the chances of planning or treatment error (12). For instance, the use of IMRT has accelerated over the past two decades; similarly, the prior decade has seen the increased use of Image Guided Radiation Therapy (IGRT). These technical advances have led to improved outcomes for many cancer patients. For instance, in a review of 30-year single institutional experience of treating base-of-tongue carcinoma, Chen et al. (29) showed improved local control (HR = 3.2), disease free survival (HR = 3.4), and overall survival (HR = 3.0), associated with the use of IMRT compared to conventional RT. Similarly, our Australian counterparts have reported functional outcome improvements in oropharyngeal cancer patients treated using IMRT compared to 3DCRT (30). Moreover, Quality of Life parameters too were shown to have improved in a randomized study using IMRT compared to 3DCRT (31). Similar outcome betterments have been reported in neuro-oncology, gynecologic oncology, urologic oncology, soft tissue sarcomas, and pediatric oncology (32–36).

There is no doubt then that IMRT/IGRT is the commonly accepted and preferred form of radiation treatment for a majority of curative treatments with radiation therapy in the USA today. Generally, though, IMRT/IGRT treatment planning and delivery is more complex and “less forgiving” of errors made in the outlining of target volumes and normal tissue contours since the dose fall-off is steep and rapid. The likelihood of errors are thus increased compared to conventional RT, and may, sometimes, be catastrophic as inaccuracies in target tissue definitions can result in their consequential under-dosing while errors in contouring normal tissues may lead to over-dosing during treatment (12, 37–39).

In this regard, several studies have documented the increased chance of errors in the defining of target volumes in the modern practice of radiation oncology, where the reliance on complex imaging modalities to outline the targets is essential in the majority of treatment plans. For instance, the inter-observer variability in treatment volume delineation was recently reviewed by Segedin and Petric, Vinod et al., and Cyran et al. All these authors reported substantial inter-physician variability in defining the target volume and strongly recommended the use of disease specific guidelines and protocols, multimodality imaging, continuing education, and auto contouring tools as ways to reduce the uncertainty in defining treatment volumes (40–42). Thus, it is becoming increasingly apparent that:

1. With the increased complexity of radiation therapy treatment planning and delivery and increased dependence on imaging in outlining treatment target volumes, the potential for errors has increased.
2. Minimizing errors is even more important now with the increased use of IMRT and IGRT in treatment delivery.

Although, many of the process-related errors associated with IMRT and IGRT can be prevented by following the American Association of Physicists in Medicine (AAPM) guidelines (see elsewhere in this paper), the “cognition-related errors” which may happen can be prevented only by adequate peer review; by “cognition-related errors” we mean mainly the physician's errors in target volume and normal tissue volume definitions. Thus, to optimally “chase zero harm” in our technology-driven specialty, we believe that a multi-disciplinary peer review of the physician-conceptualized treatment target volumes (e.g., GTV, ITV, CTV, and PTV), the normal tissue contours, and the treatment plan generated with this information, is absolutely essential. The ideal time to do this would be prior to initiating the patient's treatment since making corrections to an already initiated treatment plan can lead to increased dosimetrist workload, unanticipated treatment breaks, and decreased efficacy of the radiation treatments. Before we delve deeper into the concept of a pre-treatment peer review, we will review some of the current ways and means of peer review and quality assurance in radiation oncology, looking at where they work well and where there is room for improvement.

### Peer Review in Radiation Oncology

A direct quote from Hendee and Herman's paper from 2010 summarizing the recommendations of a multidisciplinary meeting on, “Safety in Radiation Oncology,” crystalizes our central thesis: “A single error that harms a radiation therapy patient is one error too many” (43). This statement underpins the importance of “Chasing Zero Harm” in radiation oncology. Traditionally though, the first line of defense against errors in radiation therapy has always been the physician. He/she plays a key role in what has been previously described as the four major sub-processes of radiation therapy—consultation, simulation, treatment planning, and treatment delivery (44). Although the direct involvement and presence of the physician in these processes gradually decreases as the patient proceeds sequentially through them, their supervision of the processes continues in the background. While several failure modes in radiation oncology are described in the simulation, treatment planning, and delivery processes, these tend to be generally “technical” or “process oriented” and lend themselves more appropriately to resolution and management through quality assurance/quality control or process control systems (45, 46). Unlike these activities ensuring the accuracy of the “technical” aspects of radiation therapy, a patient-specific peer review program is tasked with improving the safety and quality of the “professional” decisions made by members of the clinical radiation oncology team—decisions that may not always be clearly correct or clearly incorrect (45). Such a process is also acquiescent of the possibility of errors arising from a physician's clinical decisions and that multiple levels of review improve the likelihood of detecting and correcting the error(s) before they become incorporated into the patient's treatment.

Many if not all of the safety conference's recommendations are still relevant and are listed in Table 4 (43). However, one major criticism of the Hendee and Herman paper was that, whereas there was a heavy focus on treatment machine and quality assurance processes involved with the machines, it did not sufficiently emphasize those aspects of radiation therapy involving patient flow and processes. These would include, for instance, treatment decision making, the importance of multidisciplinary oncology team communication, treatment planning, and review of resultant treatment plans, and peer review (45).

**Table 4:** Recommendations from “Safety in Radiation Oncology” adapted with comments (43).

**Recommendation**	**Salient points (if applicable)**	**Comments**
1. As the complexity of treatment devices increases, control over the devices should be simplified.	The points made under this recommendation focuses on the treatment machine items than on planning tools or decision making issues.	The complexity of treatments has increased since 2010 and so this recommendation is even more applicable now.
2. Radiation therapist workstations should be designed according to principles of human factors engineering.		Still holds true.
3. Return control to the point of care.	Radiation Therapists should have more control over decision making at the machine.	In our department, radiation therapists are full members of the decision making and QA teams.
4. Provide improved early warnings.	This again focuses on the machine design and early warnings that can prevent machine malfunction.	Vendors have improved the machine designs since 2010.
5. Vendors should quickly and intelligibly address concerns reported by physicists and other members of the treatment team.		Still holds true.
6. User Groups.	User groups of equipments to improve communications between vendors and users about safety issues.	Still holds true.
7. The billing process should be simplified, and the radiation therapist should not be burdened with billing duties while overseeing patient treatments.		Billing processes have become more complex, not less.
8. Develop recommended staffing levels.	ASTRO and other organizations were tasked to do this.	This is even more important now with more use of IMRT, SBRT and IGRT.
9. Radiation therapy facilities should employ techniques such as failure mode effects analysis (FMEA) to identify potential sources of error and root-cause analysis (RCA) to identify and correct errors when they occur.		Still holds true.
10. Error reporting systems should be developed in radiation therapy.		This is happening now.
11. A covenant and commitment to safety should be expected of the treatment team.		Still holds true.
12. Any member of the treatment team can declare a Time Out.		Still holds true. [see later part of this paper]
13. Checklists should be employed.		Still holds true.
14. Audits should be performed.		Still holds true.
15. Facility accreditation should be attained.		This has become a more common practice now.
16. Standard operating procedures should be available and revised as necessary.		Still holds true.
17. Patient safety should be a competency.		To the best of our knowledge, this has not been implemented.
18. Safety champions should be present.		Still holds true.
19. Treatment team qualifications must be consistent and recognized nationally.		Still holds true.
20. The FDA review process should be improved.		Still holds true.

In this respect, Marks et al. later addressed the peer review aspects of radiation oncology *per se*. Although their White Paper emphasized the importance of the non-technical aspects that needed to be reviewed, even they did not fully address the importance of a pre-treatment peer review approach, although they noted the value and necessity for a multidisciplinary team—physicians, physicists, dosimetrists, and therapists—in the peer review process (45).

In a more recent publication, Huo et al. (47) also reviewed the current status of peer review in radiation oncology and noted the following:

- The importance of peer review is well accepted by radiation oncologists.
- The comprehensiveness of its implementation is less than what is desired.
- In North America, 70–80% of RT courses undergo some type of peer review (mostly after the treatment course has started and during the “chart rounds”).
- Pre-treatment peer review is emphasized in radiation oncology practice in Canada; yet, even with the emphasis, it occurs in <40% of cases.
- The overall percentage of changes in treatment plans associated with the performance of these processes is about 11%.

Brunskill et al. identified similar findings when they reviewed the “peer review of radiation plans” (48). A total of 11 publications were combined for their review and they reported that about 11% of the 11,491 cases reported in these publications required some type of treatment plan modification; about 7% of the modifications were considered “minor” and about 2% were “major.” The most common major change was target volume delineation (45%), followed by dose prescription changes (24%) for minor changes; normal tissue delineation related modifications were required in about 8%.

### Medical Physicists and Clinical Quality Assurance

Uniquely in radiation therapy practice, medical physicists play a critical role in ensuring patient safety including calibration of treatment machines, setting up the correct parameters of treatment machines in the treatment planning system, verifying the often computer automated delivery of radiation dose, certifying delivery of the correct dose to the patients, overseeing radiation safety, plus many other quality assurance (QA) tasks. The American Association of Medical Physicists (AAPM) has developed and continues to develop many guidelines in quality assurance and clinical radiation practice. To optimally chase zero harm to the patient, the timing of the performance of some of the quality assurance tasks is important. For instance, the commonly accepted practice of the initial check of a treatment chart by a medical physicist as specified in the Task Group 40 (TG40) report titled, “Comprehensive Quality Assurance for Radiation Oncology,” is for it to be performed, “before the third treatment fraction following the start of each new treatment field or field modification.” However, if through such quality assurance efforts an error is identified just before the third fraction, the damage, however small, may already be done during the treatment fractions delivered up to then. By then it is probably too late to chase zero harm in regards to that specific patient. To prevent this possibility, our institutional policy has been that the initial chart check will be performed before the first treatment fraction is delivered, regardless of the type of treatment plan—complex or simple.

It is also true that the AAPM's TG reports tend to focus more on the physics-related aspects of quality assurance rather than the medical questions related to the patient (for instance, questions such as—is the decision to treat appropriate or what should the prescription dose be?) (49, 50). As pointed out in the TG 100 report, the majority of the errors that occur in radiation oncology are not due to failures in devices or software—rather they are the consequences of failures in workflow and process (51). Although physicists do thorough and excellent quality assurance of the hardware and software of radiation oncology, mistakes in target and critical structure delineation, underdoses to the target volume, or overdoses to critical structures cannot be detected by any patient-specific QA measurements performed by the physicists, regardless whether they are performed before treatment starts or by the third fraction of treatment. Thus, incorporating a pre-treatment peer review process that is focused on the medical decision making processes that are not addressed by the quality assurance procedures of the physicists can be considered as a step further in “process improvement” for the radiation oncology department.

### Current Practice of Chart Rounds

“Chart rounds” is the most common clinical intra-departmental “peer review” process unique to radiation therapy. Other examples of individual- or multi-institution peer review processes include prospective tumor conferences, central reviews such as those conducted as part of national or international clinical trials, and programmatic and practice review programs such as those performed by the Accreditation Council for Graduate Medical Education (ACGME) or the American College of Radiology (ACR), respectively (52–54).

During chart rounds, the chart of every patient who has initiated radiation treatment is thoroughly reviewed by physicians, physicists, and representatives of the treatment team comprising nurses, therapists, and dosimetrists. This type of review is usually performed on a weekly basis and includes all the patients who had started therapy since the previous chart rounds conference. The precise logistics of this process varies from institution to institution, although there are areas of both consensus and variability. For instance, some utilize paper charts while others may use an electronic chart or a combination of both. The review includes all the data related to, but not necessarily limited to, the diagnosis, staging, treatment site, treatment intent, treatment volumes, treatment dose, dose fractionation, type of treatment plan, port films, et cetera. Commonly utilized treatment “record and verify” software for radiation therapy such as Mosaiq® or Aria® have specific modules with checklists within them which facilitate the complete review of all the items mandated by a department's leadership as being essential for a complete clinical overview of the patient's radiation therapy chart. Worldwide too, similar protocols are used in radiation therapy departments; for instance, the Royal Australian and New Zealand College of Radiologists (RANZCR) utilizes an written “audit tool” to check and record the quality of radiation therapy notes and prescriptions during their chart rounds (55, 56).

However, although its goals are comprehensive and laudatory, this conference only retrospectively identifies any shortcomings and recommends remedial actions, as it occurs after a patient has started treatment. Moreover, the time limitations in these chart rounds do not usually permit more in-depth review of the patient's treatment plan, especially if it is complex. Hence, the review of complex radiation treatment plans involving the use of IMRT, IGRT, or volumetric modulated arc therapy (VMAT), where discussion of the treatment volumes and overall plan quality would provide optimal support and guidance from radiation oncologists, both to peers and trainees, is usually beyond the scope of a traditional chart rounds.

To overcome this shortcoming and to complement the retrospective peer review of chart rounds, others and we have recommended prospective, pre-treatment group conferences to review critical and/or complex radiation treatment plans so that physicians other than the treating physician can be also involved in evaluating the quality of the plan and identifying potential errors (17, 57). Although such additional conferences are firmly established in our department and may be an evolving process in others, its purpose is separate and not specifically designed to supplant chart rounds which is in and of itself a valuable tool and provides systemic redundancy to identify errors that may have been overlooked.

### Rationale for *Pre*-treatment Peer Review

It was not long after a patient suffered serious harm during treatment—harm that was reported in the national press—that official recommendations began to strongly endorse the performance of patient-specific IMRT QA prior to the first treatment fraction rather than within the first 3 fractions of treatment (58, 59). It was tacit acknowledgment in radiation oncology that not only does the method of quality assurance matter, but also the time point of its implementation in the workflow of the clinic. Interestingly, IMRT QA has been identified as a weak quality assurance check in comparison to others such as peer review, but peer review is not as oft recommended as a pre-treatment step in the workflow (60–62). Peer review has been identified as an important and even essential component of a “high reliability organization,” but its implementation pre-treatment has not yet been identified as such, though some have tried to argue in this direction (63–66).

Looking at the reality of today's complex radiation clinical practice, however, we argue that for “high-reliability,” peer review performed early on during treatment may be acceptable, but for “zero harm,” its performance prior to the first treatment is essential. The national press article referenced earlier clearly established that any errors caught during the first week of treatment by peer review can only lead to “mitigated” rather than in “prevented” consequences (9, 11, 64, 66). Additionally, some types of errors which may not be detected through other quality assurance checks may be more recognizable during peer review, especially a peer review model which utilizes the skillsets of multiple disciplines, such as ours.

In addition to those mentioned previously, another AAPM TG report, still under development, and tentatively titled, “Best Practices for Physics Plan and Chart Review,” includes a failure-mode-effect-analysis of radiation events/errors which could be caught during the chart/plan review process. The highest scoring item recorded by this TG so far has been, “wrong or inaccurate MD contours.” This umbrella description may include any of a plethora of mistakes such as un-reviewed contours, dose levels not identified, wrong images, incorrect image fusion, lack of consideration of target motion, or even an incorrect set of contours imported from another treatment planning or review system (67). The TG not only identified these high scoring error modes, but also espoused the value of a peer review and treatment plan check occurring prior to the first treatment (67, 68). Though the physics plan check has been identified as an extremely effective quality assurance step in the workflow, the high scoring errors identified by this Task Group may not always be detectable by the physics personnel alone; hence the need for effective pre-treatment clinician peer review prior to treatment (60). In addition, target volumes and planning risk volumes often incorporate physics concepts and considerations, highlighting the value of the multi-disciplinary approach to peer review.

Finally, the personal experience of the authors of this manuscript corroborates the need for a pre-treatment peer review culture to achieve the “zero harm” goal in radiation therapy. Briefly, in 2002, one of the authors of this paper conducted a root cause analysis of 12 major errors identified in an academic radiation oncology department. The process revealed that a pre-treatment peer review and a second check of the calculations by a physicist before the first treatment delivery would have avoided all of the errors (Vijayakumar, 2002, Personal Communication). These processes were subsequently implemented within that department and no major errors were detected over the next 5 years. We too have had a similar policy in place at our institution since 2007 with correspondingly reportable success over the past 10 years [(17–19), Vijayakumar, 2018, Personal Communication)].

### Challenges of *Pre*-treatment Peer Review

Perhaps the most obvious challenge with pre-treatment peer review is that of the time investment from all participants. Allowing leeway between the scheduled start of treatment and the occurrence of peer review can alleviate some of the time burden. Requiring more than one additional physician and especially multi-disciplinary adherence in the process amplifies this time cost. This has been recognized as a challenge by many authors, but solutions have been offered as well (17, 18, 48, 62, 64, 69–73). Indeed, widespread and equitable commitment to the use of this process can be a major contributor to increasing the feasibility of its implementation. It has been our experience though that when committed, department faculty and staff will make the necessary sacrifices and provide the accountability necessary by use of their clinical schedules and communication to ensure that all patients who need to will undergo their pre-treatment peer review (17).

Once the department has committed to the performance of this process, the challenge then moves to reduction of the burden on the staff. Solutions to this vary, but may include automation of data collection and presentation, reasonable expectations for timely patient starts based on optimal timetables for patient outcomes, and defined criteria for the performance of pre-treatment peer review, even going so far as to allow some portions of the peer review to occur separately such as reviewing contours first and the treatment plan later (18, 74, 75). Ultimately, human capabilities are limited and we may see this type of peer review become more and more automated through the use of tools like machine learning and artificial intelligence. For instance, a lung radiation oncologist would have to read 8 papers per day to stay current on peer-reviewed evidence, but the horizon looks promising with decision support systems being developed that are actively tapping thousands of peer-reviewed research articles as well as current clinical data not yet published (76). IBM's Watson is a great example of movement in this direction (77). Additionally, we will likely be taking into account many more factors into treatment decisions, many of which may be invisible or unfathomable for humans. Radiomics and genomics are great examples of these factors (77). For now, however, the most reliable “peers” are our clinical colleagues and we must find ways to utilize them effectively in the pre-treatment phase in order to truly make progress toward a “zero tolerance” attitude toward errors (78, 79). If one could look ahead and know that a major error was going to occur, likely there would be no cost that one would not be willing to pay in order to prevent that error. Perhaps, we must make that assumption when we identify procedures such as pre-treatment peer review that would be effective at preventing errors, but indeed may induce more burden on staff.

### The Importance of Safety Culture in Radiation Oncology

Developing any approach to minimize errors and maximize patient safety starts with implementing a culture of safety. Institutions inside and outside healthcare strive to be highly reliable and efficient. Any healthcare culture of safety must have a focus on failures and near misses and the courage to discover and address that “bad news.” While building a culture requires a whole team effort, its creation must initially be catalyzed by the leadership viewing mishaps as an opportunity to learn and improve. Of course, it is always better to address a problem before it becomes a failure or near miss, but the culture needed to address problems proactively is largely the same as that needed to address problems retroactively.

The Agency for Healthcare Research and Quality (AHRQ) cites four main features of a culture of safety (80). First, an organization must have knowledge that it is engaged in high-risk activities, and it must be determined to maintain safe operations. Essentially then, there must be recognition that there is a potential for harm, and that recognition must trigger a deep motivation to actively minimize that potential.

The second feature is the establishment of a blame-free environment, in which error reporting can take place without repercussions. Crucial to eliminating errors is complete knowledge of the error, and facilitating dissemination of the knowledge derived from an error cannot happen if there is a culture of fear built around the reporting an error. Another feature outlined by the AHRQ is the unfailing commitment or the organization to address safety issues. This feature addresses the motivation of the organization, and without a strong commitment to the process from the entire team, efforts will not be fruitful. The AHRQ further encourages the collaboration across disciplines and titles to seek new and innovative solutions with regard to patient safety as a necessary feature for a culture of safety. The collaboration allows the team to collect perspectives from various points of view and produce solutions that are novel and more comprehensive (80).

The importance of a team concept is paramount in error detection and prevention. It is estimated that 70–80% of errors in medicine are the result of ineffective teamwork (81–83). Teamwork is often inhibited by hierarchy and while many institutions have begun to formally address the issues of hierarchical behavior, including education at the medical school level, this behavior is nevertheless well-established in healthcare and will take time to mitigate (84). If an organization puts a high value on developing and maintaining a culture of safety, it must also be willing to prioritize the development of a team-oriented culture without rigid hierarchy.

### “Anybody can Raise His/Her Hand”—Importance of Equality in Safety Culture

The avoidance of hierarchical issues is of paramount importance in our department's peer review process. In fact, key to our safety culture is peer review of treatment plans before the start of treatment and careful review of information for patients currently on treatment through chart rounds. These review sessions create a venue where representatives from the physicians, therapists, physicists, dosimetrists, and nurses can voice their concerns. These sessions have been successful in preventing errors and improving the quality of treatment plans because of the collaborative culture that has been established within the department. All members of the team are encouraged to comment on each plan or treatment, and because comments are made without fear of hierarchical retribution, members are more forthcoming with their genuine concerns regarding an isodose line, or a dose constraint, or even with the decision to treat. The staff members' presence at the review session also allows for increased efficiency and effective communication, due to the immediate feedback received and facilitates the discussions that ensue. In addition, the multidisciplinary team allows for a more thorough critique of a treatment plan by bringing a wide spectrum of perspectives to the analysis.

## Summary and Conclusions

“Chasing Zero Harm” is a new patient safety initiative that is endorsed and spearheaded by the JCAHO with the aim of re-creating healthcare entities as, “high reliability organizations,” that practice a form of “collective mindfulness”—i.e., a constant vigilance focused on preventing, rather than reacting to, errors in patient care. Although radiation oncology has an excellent reputation for patient safety, we still have room for improvement. Here, we have provided a brief overview of the “chasing zero harm” movement and its relation to “high reliability organizations.” We argue that all radiation oncology departments need to become HROs to achieve the goal of zero harm to our patients. In looking at our safety record in radiation oncology, we differentiate between technical processes and clinical practices and discuss the existing quality systems designed to ensure safety in both aspects—regular quality assurance processes initiated and maintained by the medical physicists and on-going physician overview of clinical workflow including treatment planning, which culminates in chart rounds, respectively. We document the strengths of these efforts in ensuring that “safe machines deliver safe treatments” and identify some of the inherent weaknesses of these processes insofar as the timing of their performance. We then introduce the concept of a pre-treatment peer review of the clinical decisions that have been made and the radiation treatment plans that have resulted and posit that its addition to the clinical workflow takes us one step further to the goal of chasing zero harm in radiation oncology. Further, we describe our 12-year experience using the pre-treatment peer review process and the lessons we have learned that were important for its success—specifically, the unrelenting commitment to a safety culture within the department, the minimization of clinical hierarchy in the peer review process, and the importance of a culture of equality as the treatment plan's quality is being debated. A recent framework publication from the American Society of Radiation Oncology emphasizes the importance of prospective peer review. They state “prospective peer review is critical because once treatment has been initiated, the threshold for making meaningful change is relatively high because of time consuming replanning and QA requirements (85).” In conclusion, we strongly believe that taking the next logical step toward zero harm in radiation oncology involves the active pursuit and implementation of a pre-treatment peer review program as we have observed our model succeed in both preventing errors and improving the quality of our patients' treatment.

## Author Contributions

SV conceptualized and oversaw the work as well as assisting in the manuscript drafting. WD, SP, BM, and CY all contributed various portions of the manuscript draft and literature review. All authors read and approved the final manuscript.

### Conflict of Interest Statement

The authors declare that the research was conducted in the absence of any commercial or financial relationships that could be construed as a potential conflict of interest.

## Abbreviations

GCPR: Group Consensus Peer Review
IOM: Institute of Medicine
JCAHO: Joint Commission for Accreditation of Healthcare Organizations
HRO: High Reliability Organization
RPI: Robust Process Improvement
IMRT: Intensity Modulated Radiation Therapy
3DCRT: Three Dimensional Conformal Radiation Therapy
IGRT: Image-Guided Radiation Therapy
QA: Quality Assurance
AAPM: American Association of Physicists in Medicine
TG: Task Group
ACGME: Accreditation Council for Graduate Medical Education
ACR: American College of Radiology
RANCZR: Royal Australian and New Zealand College of Radiologists
VMAT: Volumetric Modulated Arc Therapy
SBRT: Stereotactic Body Radiation Therapy
DVH: Dose Volume Histogram
GTV: Gross Tumor Volume
CTV: Clinical Target Volume
ITV: Internal Target Volume
PTV: Planning Target Volume
AHRQ: Agency for Healthcare Research and Quality.
